# Quercetin potentializes the respective cytotoxic activity of gemcitabine or doxorubicin on 3D culture of AsPC-1 or HepG2 cells, through the inhibition of HIF-1α and MDR1

**DOI:** 10.1371/journal.pone.0240676

**Published:** 2020-10-14

**Authors:** Sarah Hassan, Jean Peluso, Sandra Chalhoub, Ysia Idoux Gillet, Nadia Benkirane-Jessel, Natacha Rochel, Guy Fuhrmann, Genevieve Ubeaud-Sequier

**Affiliations:** 1 Regenerative Nanomedicine, INSERM UMR 1260, FMTS, University of Strasbourg, Strasbourg, France; 2 Platform eBiocyt-UPS1401, Faculty of Pharmacy, University of Strasbourg, Strasbourg, France; 3 Department of Integrative Structural Biology, Institut de Génétique et de Biologie Moléculaire et Cellulaire, INSERM, U964 CNRS UMR 7104, Université de Strasbourg, Strasbourg, France; 4 Department of Pharmacy, Strasbourg University Hospital, Strasbourg, France; Sechenov First Medical University, RUSSIAN FEDERATION

## Abstract

The impact of cancer on lifespan is significantly increasing worldwide. Enhanced activity of drug efflux pumps and the incidences of the tumor microenvironment such as the apparition of a hypoxic gradient inside of the bulk tumor, are the major causes of chemotherapy failure. For instance, expression of Hypoxia-inducible factor (HIF-1α) has been associated with metastasis, resistance to chemotherapy and reduced survival rate. One of the current challenges to fight against cancer is therefore to find new molecules with therapeutic potential that could overcome this chemoresistance. In the present study, we focused on the bioactive plant flavonoid quercetin, which has strong antioxidant and anti-proliferative properties. We examined the efficacy of combined treatments of quercetin and the anti-cancer drugs gemcitabine and doxorubicin, known to specifically act on human pancreatic and hepatic cancer cells, respectively. Moreover, our study aimed to investigate more in-depth the implication of the multidrug transporter MDR1 and HIF-1α n chemoresistance and if quercetin could act on the activity of the drug efflux pumps and the hypoxia-associated effects. We observed that the anti-cancer drugs, were more effective when administered in combination with quercetin, as shown by an increased percentage of dead cells up to 60% in both 2D and 3D cultures. In addition, our results indicated that the combination of anti-cancer drugs and quercetin down-regulated the expression of HIF-1α and increased the expression levels of the regulator of apoptosis p53. Moreover, we observed that quercetin could inhibit the efflux activity of MDR1. Finally, our in vitro study suggests that the efficiency of the chemotherapeutic activity of known anti-cancer drugs might be significantly increased upon combination with quercetin. This flavonoid may therefore be a promising pharmacological agent for novel combination therapy since it potentializes the cytotoxic activity of gemcitabine and doxorubicin on by targeting the chemoresistance developed by the pancreatic and liver cancer cells respectively.

## Introduction

A variety of phytochemicals with promising anticancer potential has been tested against human cancer [1]. In particular, one member of the polyphenol family, namely quercetin (3,5,7,3’,4’-Pentahydroxyflavone) has been intensively studied for its anticancer properties [2]. This flavonoid is characterized by the presence of five hydroxyl groups on C6-C3-C6 backbone structure, especially a 3-OH group on the pyrone ring [3]. The beneficial effect of quercetin has been reported in various diseases since it displays anti-cancer, anti-inflammation and antioxidant effects [4]. For instance, quercetin has been shown to inhibit the expression of nuclear factor-kappa B-dependent inflammatory genes. Moreover, it inhibits the proliferation of cancer cells through the activation of various apoptotic signals. However, previous studies suggest another mechanism for its ability to suppress cancer metastasis, independently of its properties to induce cell death [5].

Hypoxia is a common condition in solid malignancies where cells proliferate under a low pressure of oxygen and nutrients [6]. This process implicates the activation of hypoxia-inducible factor 1 α (HIF-1 α) which is one of the most important proteins involved in the outgrowth and metastasis of cancer cells [7]. Indeed, the transcription factor HIF-1α regulates many genes associated with cell survival and migration, oxygen transport, angiogenesis, and glucose transport [8]. Various natural products that can down-regulate HIF-1α have been identified, such as luteolin [9]. Furthermore, several phytochemicals with a known antioxidant activity can down-regulate the expression of HIF-1α, which is known to be associated in cancer with increased levels of reactive oxygen species (ROS) [10]. Actually, this accumulation of ROS results from a mitochondrial dysfunction and increases the metabolic activity of the cancer cell [11].

One of the first report on the molecular mechanisms explaining the contribution of hypoxia to drug resistance was the finding that HIF-1α is able to activate the multidrug resistance MDR1 gene [12]. It is therefore highly valuable to find novel ways to block, in tumor cells under hypoxic stress, the expression of HIF-1α and consequently, that of MDR1.

Indeed, the major cause of chemotherapy failure is the resistance developed by the cancer cell to anticancer drug [13]. An overexpression of MDR1 has been shown to be involved in this process. MDR1 acts as an energy-dependent drug efflux pump, thereby decreasing the intracellular drug concentration and causing drug resistance [14]. For example, colorectal cancer cells express high levels of MDR1, which is known to contribute to their general resistance to anticancer drugs. The very limited efficiency of chemotherapy for cancer patients is therefore associated with the inherent chemoresistant nature of this aggressive disease [15]. To enhance the anticancer therapeutic efficacy and reduce the side effects, it has been suggested that natural products can be combined with standard chemotherapy and radiotherapy [16]. Many studies with polyphenols, such as flavonoids from fruits and vegetables, have shown that they are efficient chemopreventive agents since they are able to hamper the development of various types of cancer cells [17]. To enhance the intracellular anticancer drug accumulation by impairing the MDR1 drug efflux function, the typical process of an efficient chemotherapy involves the co-administration of a MDR1 inhibitor with an anticancer drug.

Drug inactivation, increased drug efflux and the involvement of the tumor heterogeneity and microenvironment contribute to chemoresistance. From this point of view, the diversity within the tumor microenvironment, in terms of the amount of oxygen available and acidity, plays a fundamental role [18]. To study the impact the tumoral microenvironment, it has been demonstrated that the three-dimensional (3D) cell culture model is able to recapitulate the structure, organization, and functionality of *in vivo* tissues. Cells in 3D environment have very different behaviors, and in particular for their responses to drug administration, in comparison to cells on two-dimensional (2D) cell models [19]. Starting from simple 2D monocultures, the complexity has historically increased stepwise to include stromal cells in 2D co-cultures, and in a meet step, the cultures in 3D have reached a growing interest [20]. It is now strongly recommended to upgrade cell culture from 2D to 3D models [21], since they present differences in gene expression levels and sensitivity to chemotherapeutic agents. These differences are related to the specific interactions with surrounding cells and the physical constraint between cells that affect the expression of genes and proteins, including surface receptors [22].

In the present study, we show that quercetin could sensitize pancreatic and liver cancer cells cultured in 3D culture to respectively gemcitabine and doxorubicin, which are the current anticancer drugs known to target them. Additionally, we demonstrate that the combination of these drugs induces increased cell death by inhibiting the expression of HIF-1α and the activity of MDR1. This study highlights therefore the specific properties of quercetin, which could be a promising candidate for efficient combination chemotherapy.

## Materials and methods

### Chemicals and drugs

Rhodamine 123 (RH 123) was purchased from Invitrogen (Cergy Pontoise, France). Verapamil, quercetin, doxorubicin, and gemcitabine were purchased from Sigma-Aldrich (Saint-Quentin Fallavier, France). Final concentration of DMSO applied to cells during incubation with tested drugs was 0.5%. In the tested setup, this concentration had no adverse effects on cell viability, and cell morphology or on rhodamine-123 efflux.

### Cell culture and maintenance

The human pancreatic adenocarcinoma cell line AsPC-1 (CRL-1682) and the human hepatocellular carcinoma cell line HepG2 (CRL-8065) were obtained from the American Type Culture Collection (LGC Standards, Molsheim, France). These cells were cultivated in the physiological nutrient-rich DMEM-based media (Sigma-Aldrich) supplemented with 10% (v/v) foetal bovine serum (Lonza, Verviers, Belgium), 2 mM glutamine, penicillin (100 unit/ml and 100 μg/ml) (Sigma-Aldrich). Cells were grown in petri dishes to 70–80% confluency prior to treatment. All plates were incubated in a humidified incubator at 37°C and 5% CO2.

### 3D organotypic spheroid culture

The culture of multicellular spheroids was accomplished on the GravityPlus system (InSphero AG, Zürich, Switzerland). The micro-tissues were transferred into the 96-well GravityTrap plates (InSphero AG) for toxicity studies.

### Detection of apoptosis by annexin-FITC assay

Cells death was assessed using AnnexinV-FITC Kit (Miltenyi Biotec, Paris, France) according to manufacturer’s protocol. Briefly, AsPC1 and HepG2 cells were respectively incubated with the gemcitabine and doxorubicin alone, or in combination with quercetin for 24 hours. Cells were then washed with phosphate buffered saline (PBS) and stained with AnnexinV-FITC and propidium iodide (PI). The fluorescence intensity of AnnexinV-FITC stained cells at 530/540 nm and PI stained cells at 675/630 nm were analyzed by Guava EasyCyte Plus capillary flow cytometer (Merck Millipore, Darmstadt, Germany) and computed using the Guava ExpressPro software (Merck/Millipore). The apoptotic potential of the tested drugs was compared to the apoptotic potential of celastrol (Sigma-Aldrich), which was used as positive control.

### MDR-1 functional assay

Flow cytometric measurement of MDR1 functional activity was carried out by using rhodamine 123 efflux assay. This fluorescent dye is a substrate for MDR1 and its transport out of the cell has been demonstrated to reflect MDR-1 activity. The variation in rhodamine 123 intracellular fluorescence in presence of a specific pharmacological agent is compared with that induced by a standard MDR-1 inhibitor (i.e., verapamil) which is used as positive control.

MDR1-mediated efflux of rhodamine 123 was monitored on a Guava EasyCyte Plus capillary flow cytometer equipped with a 488 nm excitation laser. The accumulated intracellular fluorescence intensity of rhodamine 123 at 530/540 nm was computed on the Guava ExpressPro software in terms of x-geometric mean arbitrary units. Dead cells were excluded based on propidium iodide staining. The inhibitory potential of tested compounds on rhodamine-123 efflux was expressed relative to maximum inhibition obtained with 100 μM verapamil in the same experiment.

### Western blot analysis

AsPC-1 and HepG2 cells were incubated for 24 hours with gemcitabine and doxorubicin respectively, in presence or absence of quercetin. Cells were collected after trypsinization and transferred to 15 ml tubes and centrifuged at 500 *g* for 5 minutes. Cell pellets were washed three times with cold 10 mM Tris- HCl, pH 7.4, 150 mM NaCl, and solubilized in lysis buffer (RIPA) (150 mM NaCl, 1% NP-40, 0.5% deoxycholic acid, 0.1% SDS, 0.5 M Tris pH 8.0). After spinning for 20 minutes at 10,000 *g* at 4°C, protein concentrations were determined in supernatants using Bradford assay. All samples were denatured by heating for 5 minutes at 95° C, before being loaded onto a 10% gel for p53, cleaved caspase 3 and HIF-1α detections. After electrophoresis, Western gels were blotted and transferred to nitrocellulose membrane which was blocked with 5% milk powder in 50mM Tris-HCl, 150 mM NaCl, 0.1% Tween 20 at room temperature for 1 hour. The membrane was then immunoblotted overnight at 4°C with either a rabbit monoclonal anti-HIF-1α (Cell Signalling Technology, Danvers, MA, USA), a rabbit monoclonal anti-p53 (Santa Cruz Biotechnology, Heidelberg, Germany), a rabbit monoclonal anti-cleaved caspase 3 (Santa Cruz Biotechnology), or a mouse polyclonal anti-GAPDH (Abcam, Paris, France), diluted at 1/1,000-1/10,000 in TBS tween 0.1%. Subsequently, membranes were washed three times with 50 mM Tris- buffered saline and 0.1% Tween 20. Following incubation with horseradish peroxidase conjugated anti-rabbit or anti-mouse secondary antibody diluted at 1/10,000 (Abcam) for 1 hour at room temperature, the blots were developed using ECL chemiluminescence substrate solution (GE Healthcare, Sarclay, France). Autoradiographic signals were captures on a GeneGenius imaging system (Syngene, Cambridge, UK) using the GeneSnap software and analyzed on NIH's Image J software.

### Statistical analysis

Data were expressed as means +/- standard error of mean (S.E.M.) and analysed using GraphPad Prism5 (La Jolla, CA, USA). Statistical analysis was performed with the one-way ANOVA test, followed by Student’s t-test. A P value < 0.05 was considered significant.

## Results

### Apoptotic potential of quercetin, doxorubicin, and gemcitabine

**Fig 1:**
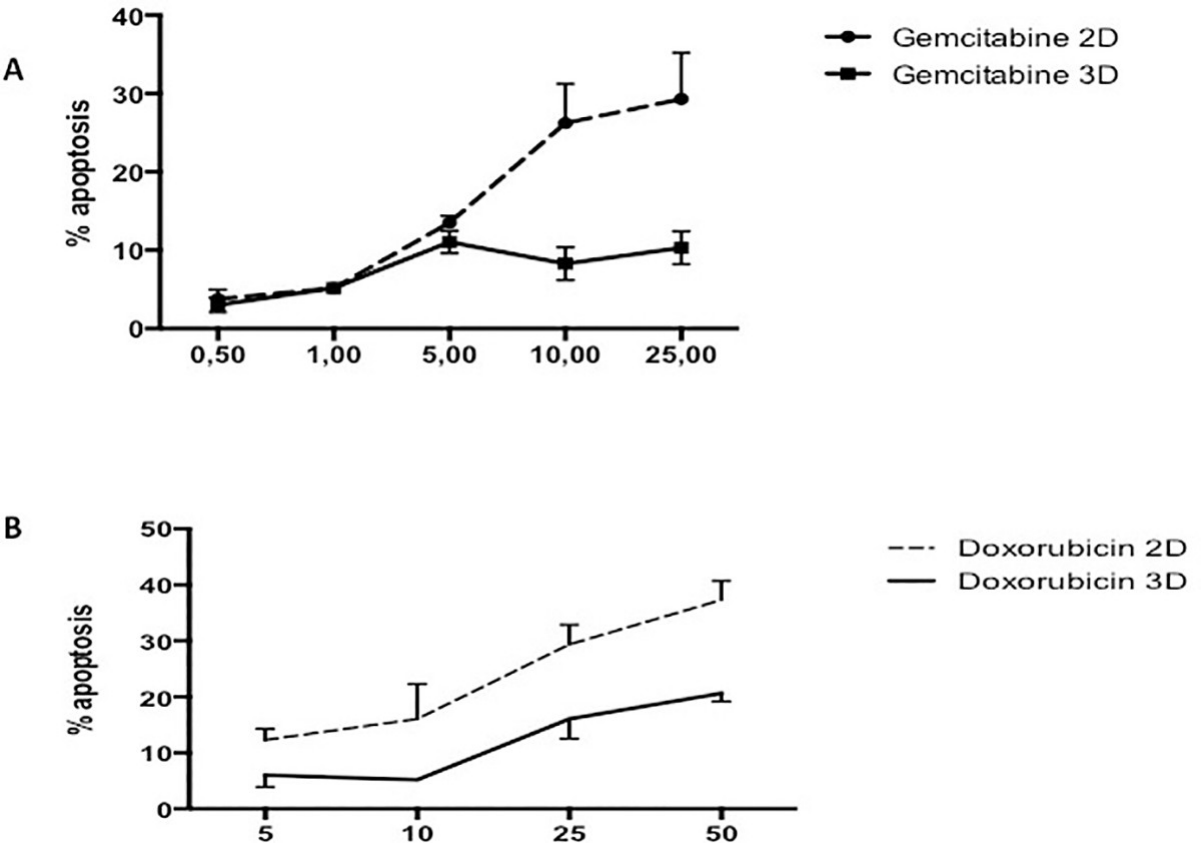
Respective apoptotic effect of gemcitabine or doxorubicin on AsPC-1 or HepG2 cells. Apoptosis rate in cells treated for 24h with the considered anticancer drug was assessed by capillary flow cytometry after staining with Annexin V-FITC and PI. (A) Recapitulates in a dose-response curve the percentage of AsPC-1 cells cultured in 2D or 3D conditions undergoing apoptosis after treatment with gemcitabine at different concentrations. (B) Recapitulates in a dose-response curve the percentage of HepG2 cells in 2D or 3D conditions undergoing apoptosis after treatment with doxorubicin at different concentrations. Data are represented as mean ± S.E.M (n = 3).

**Fig 2:**
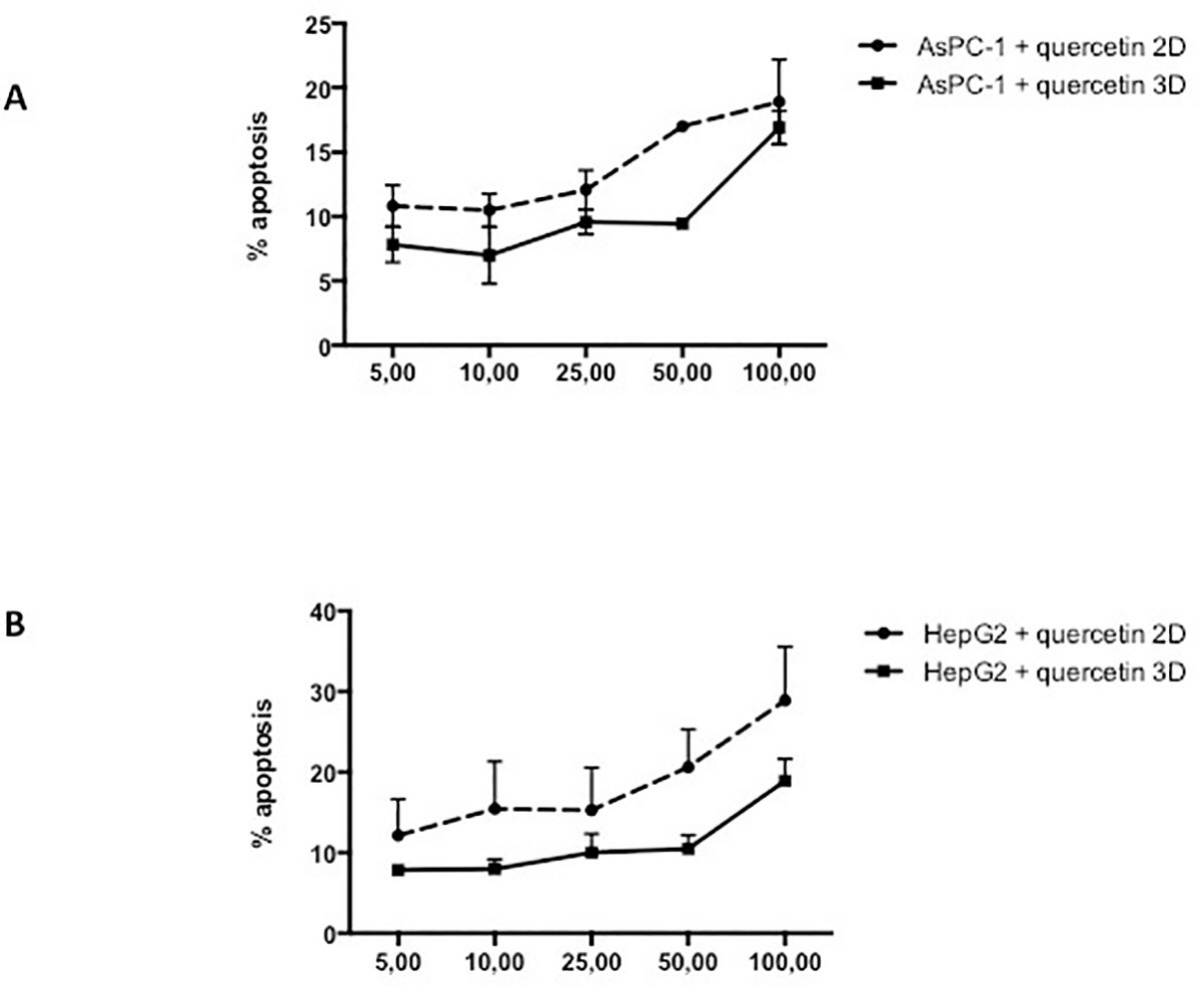
Apoptotic effect of quercetin on AsPC-1 and HepG2 cells. Apoptosis rate in cells treated for 24h with quercetin was assessed by capillary flow cytometry after staining with Annexin V-FITC and PI. (A) Recapitulates in a dose-response curve the percentage of AsPC-1 cells cultured in 2D or 3D conditions undergoing apoptosis after treatment with quercetin at different concentrations. (B) Recapitulates in a dose response curves the percentage of HepG2 cells cultured in 2D or 3D conditions undergoing apoptosis after treatment with quercetin at different concentrations. Data are represented as mean ± S.E.M (n = 3).

**Fig 3:**
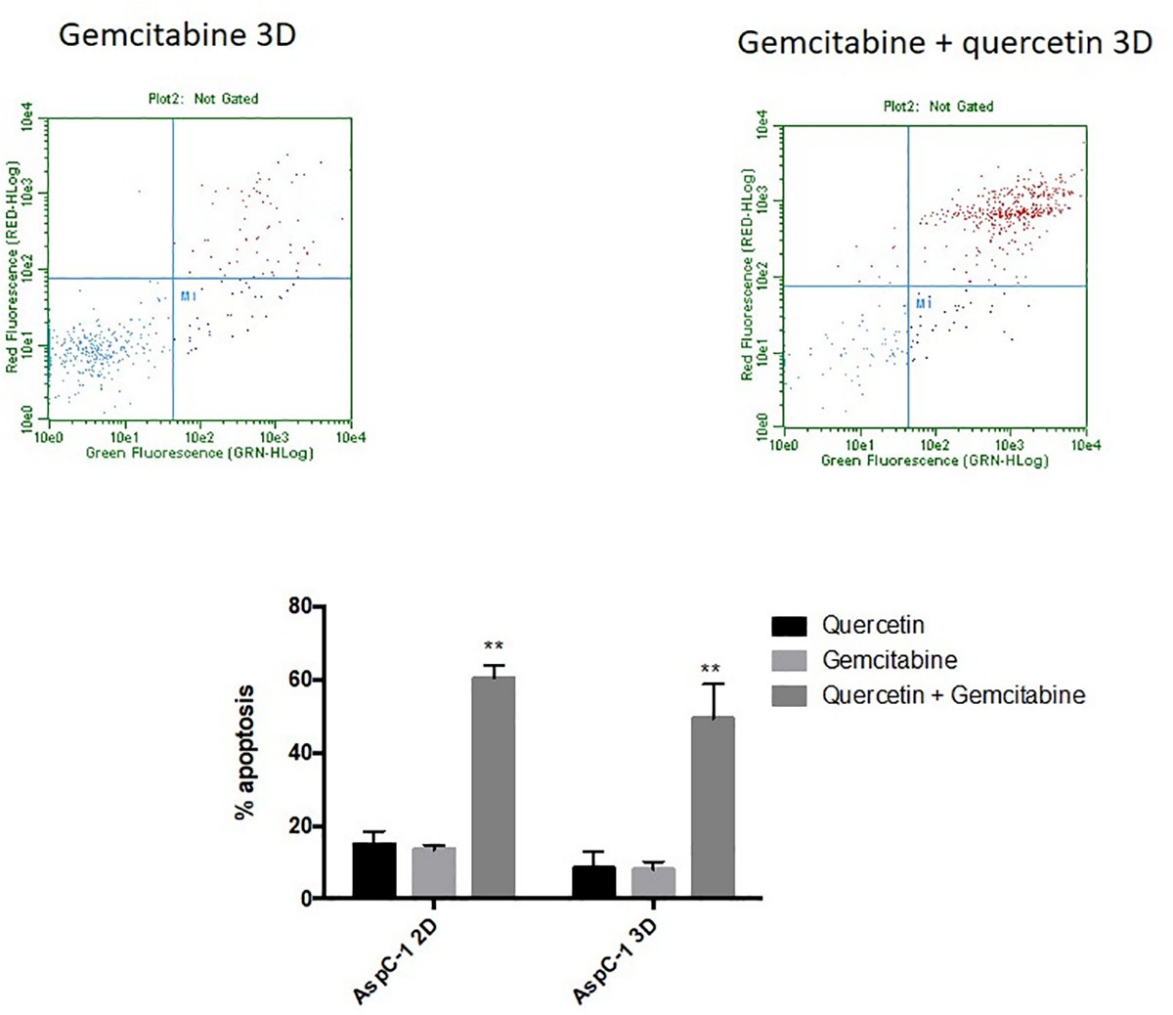
Apoptotic effect of quercetin on gemcitabine-treated AsPC-1 cells cultured in 2D or 3D conditions. Cell cytotoxicity was induced after treatment with gemcitabine at 5 μg/ml, in combination or not with 50 μM of quercetin. The upper panels show the results of representative scatter plots obtained after the different treatments; cells of the lower left quadrant are viable, cells on the upper and lower right quadrants are in late and early apoptosis, respectively. The lower panel shows the number of cells in late apoptosis, expressed as percent relative to total cell number. Data are represented as mean ± S.E.M (n = 3). **, P < 0.01.

**Fig 4:**
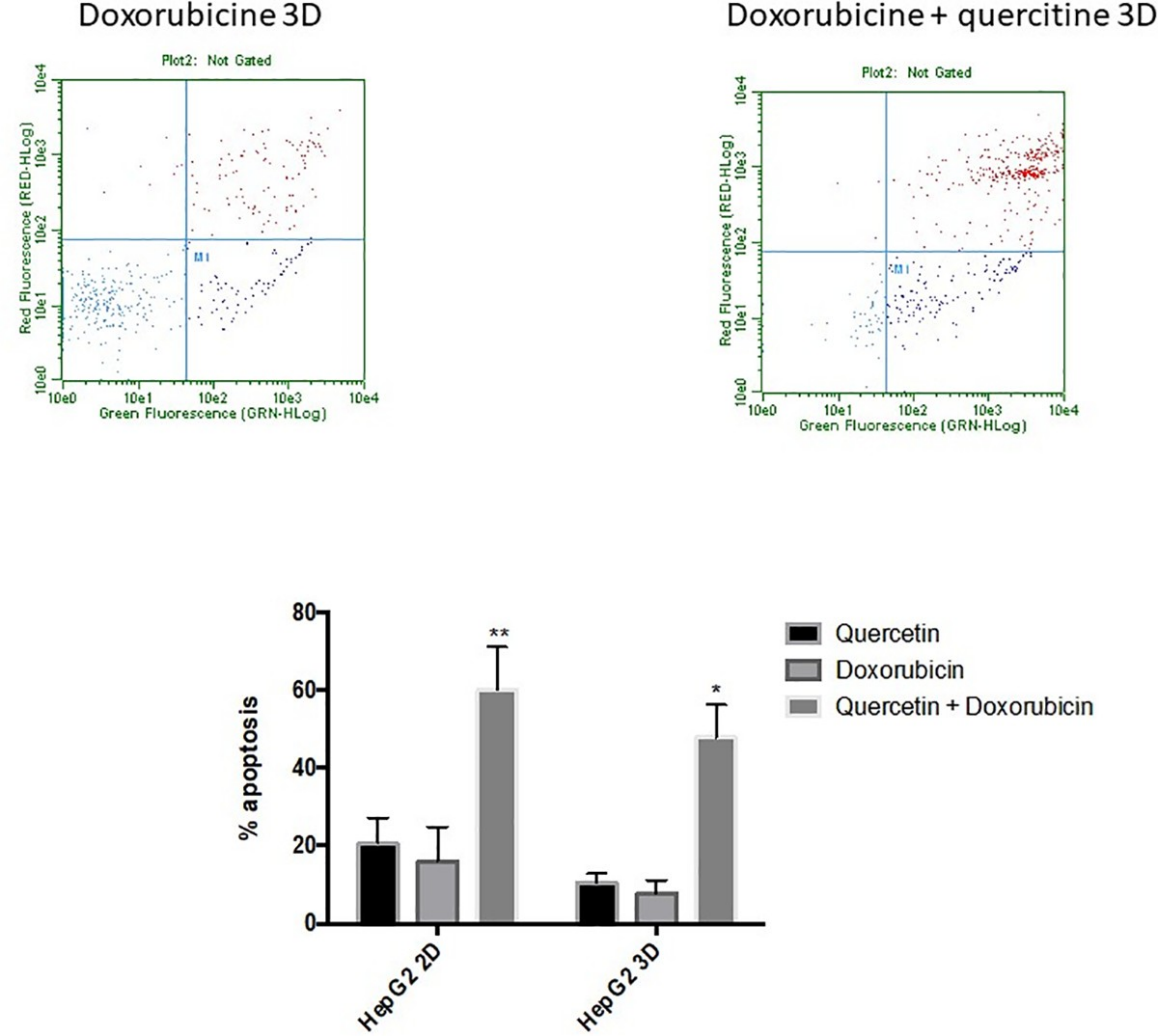
Apoptotic effect of quercetin on doxorubicin treated HepG2 cells cultured in 2D or 3D conditions. Cell cytotoxicity was induced after treatment with doxorubicin at 10 μM, in combination or not with 50 μM of quercetin. The upper panels show the results of representative scatter plots obtained after the different treatments; cells of the lower left quadrant are viable, cells on the upper and lower right quadrants are in late and early apoptosis, respectively. The lower panel shows the number of cells in late apoptosis, expressed as percent relative to total cell number. Data are represented as mean ± S.E.M (n = 3). **, P < 0.01; *, P <0.05.

AsPC-1 cells cultured in 2D and treated for 24 hours with gemcitabine only exhibit a mild apoptotic rate in the range tested (1–25 μg/ml) (Fig 1A), when compared to the negative control. Moreover, our results show an increased resistance to gemcitabine when cells were cultured in 3D culture. Likewise, administration of quercetin in the range tested (0–100 μM) does not induce cell death in AsPC-1 (Fig 2A) either in 2D or 3D culture. Based on the above results and literature, we chose 5 μg/ml of gemcitabine for subsequent experiments to test the effects of gemcitabine in combination with quercetin. In the range tested, the combined drugs exhibited significantly more potent apoptotic effects both in 2D and 3D cultures, than when administered alone (Fig 3). These results demonstrated therefore that gemcitabine synergizes with quercetin to promote a strong apoptosis in AsPC-1 cells. On the other hand, HepG2 cells, cultured either in 2D or 3D and treated for 24 hours with doxorubicin, exhibit only a mild apoptotic rate in the range tested (5–50 μM) (Fig 1A), when compared to the negative control. Moreover, administration of quercetin alone in the range tested (0–100 μM) has only a mild effect on the apoptosis rate in either 2D or 3D culture (Fig 2B). Based on the above results and literature, we chose 10 μM of doxorubicin for subsequent experiments to test the effects of doxorubicin in combination with quercetin. In the range tested, the combined drugs exhibited much more potent apoptotic effects both in 2D and 3D cultures, than when administered alone (Fig 4). The results demonstrated therefore that doxorubicin synergizes with quercetin to promote cell apoptosis in HepG2 cells, even in 3D culture.

### Quercetin effect on MDR1 activity

**Fig 5:**
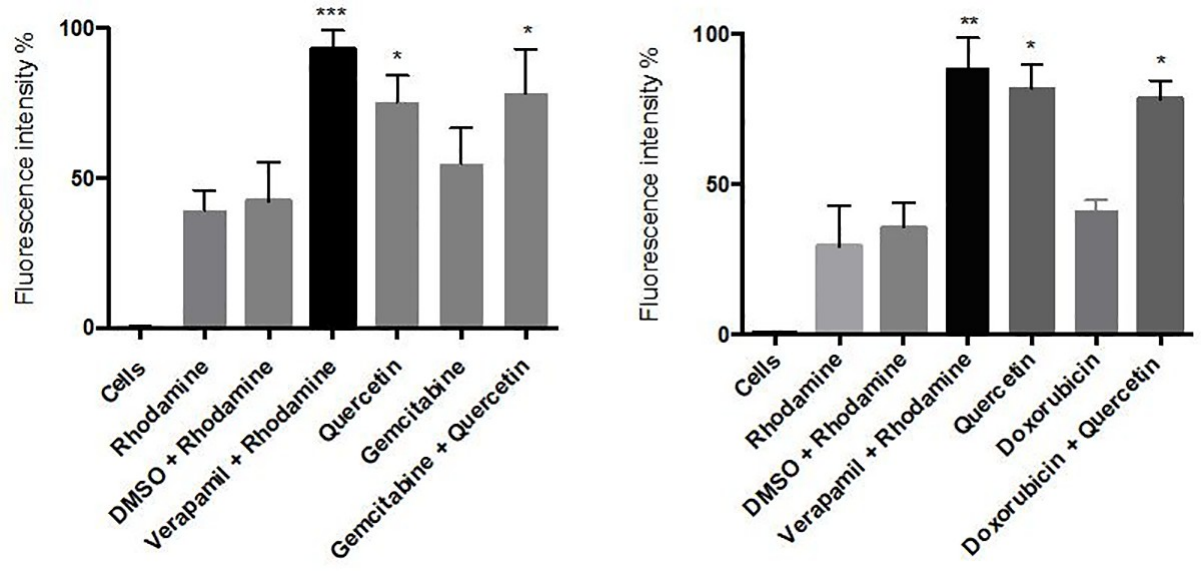
Effect of quercetin on MDR1-mediated efflux of rhodamine 123 in 3D cell cultures. AsPC-1 (A) and HepG2 (B) cells were respectively treated with gemcitabine or doxorubicin for 24 h in the presence or not of quercetin. Data are represented as mean ± S.E.M (n = 3). ***, P <0.001; **, P <0.01; *, P <0.05.

Based on above results, we chose 50 μM of quercetin to evaluate its impact on MDR1 function. As positive control, we evaluated first the effects of the specific MDR1 inhibitor verapamil on both 2D and 3D cultures. The inhibitory potential of this drug has been evidenced through an increase of the intracellular fluorescence of rhodamine 123-loaded cells, demonstrating its repressive effect on the efflux activity of MDR1 (Fig 5). We then investigated the effect of quercetin, in presence or not of gemcitabine or doxorubicin, in 3D cultures of AsPC1 or HepG2 cells, respectively. Our results showed that quercetin is able to repress the efflux activity of MDR1 in both treated or untreated pancreatic or hepatic cancer cell lines (Fig 5), suggesting that quercetin has similar properties as verapamil.

### Increased expression levels of p53 and cleaved caspase 3 after quercetin addition to gemcitabine or doxorubicin treatment

**Fig 6:**
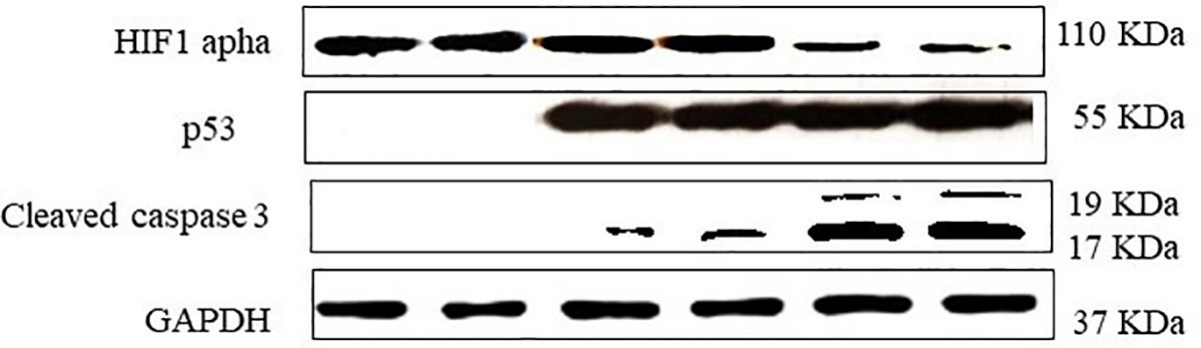
Effect of quercetin on the expression of p53, caspase 3 and HIF-1 in gemcitabine-treated AsPC-1 or doxorubicin-treated HepG2 3D cell cultures. Cells were treated with either gemcitabine or doxorubicin, in the presence or absence of quercetin. 1: Untreated AsPC-1 cells, 2: Untreated HepG2 cells; 3: gemcitabine-treated AsPC-1; 4: doxorubicin-treated HepG2; 5: gemcitabine and quercetin-treated AsPC-1; 6: doxorubicin and quercetin-treated HepG2.

Based on the above results, we chose 50 μM of quercetin, 10 μM of doxorubicin and 5 μg/ml of gemcitabine for subsequent experiments to test the effects of the considered drug combination on the expression of the apoptosis regulator p53 and the apoptosis effector caspase 3 in either AsPC-1 or HepG2 cell line. Our results showed that the expression levels of cleaved caspase 3, in particular, were significantly increased after addition of quercetin to cells treated with the anticancer drugs (Fig 6). These results indicate therefore that the combination of quercetin to either gemcitabine or doxorubicin triggers an increased apoptosis rate in pancreatic or hepatic cancer cells, respectively.

### Down-regulation of HIF-1α after quercetin addition to gemcitabine or doxorubicin treatment

Based on above results, we chose 50 μM of quercetin, 10 μM of doxorubicin and 5 μg/ml of gemcitabine for subsequent experiments to test the effects of the considered drug combination on the expression of HIF-1α in 3D culture of AsPC-1 or HepG2 cells. First, we observed that AsPC-1 and HepG2 cells cultured in 2D monolayer conditions do not express HIF-1α. In contrast, our results showed that HIF-1α is strongly expressed in 3D culture and its levels decrease when AsPC-1 or HepG2 cells are treated with a combination of quercetin and either gemcitabine or doxorubicin respectively (Fig 6).

## Discussion

In several cancer types, increased activity of the drug efflux pumps have been associated with chemoresistance, known as multiple drug resistance [23]. This hinders the efficiency of numerous anticancer drugs which are renowned to specifically target aggressive cancer types. For instance, gemcitabine has been the frontline chemotherapeutic agent against pancreatic cancer and has offered some relief over the past two decades [24]. On the other side, doxorubicin is one of the most widely used chemotherapeutic agent for treatment of human malignancies, including liver cancer [25]. However, these two anticancer drugs frequently fail to provide an effective survival benefit over the time. Actually, the National Comprehensive Cancer Network (NCCN) guidelines have highlighted the success of a therapy, which implicates the administration of a considered anticancer drug in a combination regimen [26]. For instance, recent studies indicate that natural products could be proposed for such combination strategy to treat pancreatic or liver cancer, due to their high efficacy and low toxicity [27]. Accordingly, several works have evidenced an inhibitory action of flavonoids on drug rejection, by targeting MDR1. This suggests therefore that flavonoids could increase the sensitization of cancer cells to anti-cancer drugs and therefore could enhance the chemotherapeutic process devoted to treat either pancreatic or hepatic cancer [28].

On the other hand, the fail rate in clinical trial is currently of 96% for the various anti-cancer compounds, which were previously identified in pre-clinical studies. There is therefore an urgent need to develop new pre-clinical in vitro models which could detect more accurately potential anticancer drugs [29]. For instance, 3D cell models are expected to recapitulate more precisely the response of candidate anti-cancer drugs, which could be initiated in the in vivo conditions. Indeed, 2D monolayer culture lacks to develop interaction of cancer cells with their microenvironment [30]. In contrary, many studies have demonstrated that spheroids are able to mimic the process of cancer cell metastasis [31]. Furthermore, 3D cell models are now expected to be accurate tools for the screening of promising anticancer agents, since they develop physiological cell–cell and cell–ECM interactions, mimicking therefore, in a more truthful way, the specificities of the in vivo situation [32]. In addition, one major interest on using 3D models is that dense and large spheroids develop hypoxic regions with an important expression of HIF-1α, due to the lack of nutrients and the establishment of an oxygen gradient, as also observed in vivo [33].

In a recent study, we have tested the combination of anticancer drugs with selected polyphenols, like catechin or bergamottin. Our results showed that the combination was more effective than anticancer drugs alone, since we observed increased percentage of dead AsPC-1 and HepG2 cells in 2D monolayer cultures. However, the same combinations were not effective on cells grown in 3D cultures [34]. The present work was therefore designed to study the potential of another polyphenol, namely quercetin, to respectively potentialize the cytotoxic activity of gemcitabine and doxorubicin in pancreatic and liver cancer cells, when they are cultured in 3D conditions.

In the present study, the cytotoxic properties of either gemcitabine or doxorubicin, in presence or not of quercetin, were analyzed in AsPC-1 and HepG2 cell lines respectively, in order to evaluate any supra-additional effect. MDR1-inhibiting agents are known to be pharmacologically active in vitro in a concentration range from 1 to 15 μg /ml [35]. A range of 1–50 μM of the micronutrient quercetin was therefore chosen in this study, as minimally cytotoxic doses. As expected, the combination of quercetin with gemcitabine or doxorubicin has a strong potentialization effect on the apoptosis rate of the studied cancer cell lines. Several works indicate that high expression of the drug efflux pump MDR1 is common in both pancreatic and hepatic tumours, and so, could potentially contribute, at least in part, to the chemoresistant properties of these cancers. MDR1 blockade might have therefore a very important role in the intracellular accumulation and the cellular pharmacokinetic behaviour of many anticancer drugs. Our results showed that quercetin possesses a potent inhibitory potential on MDR1-mediated efflux of rhodamine 123, when compared to the control verapamil; this suggests that quercetin is able to increase the intracellular accumulation of either gemcitabine and doxorubicin, enhancing thereby its proapoptotic effect in the long-term. Accordingly, our study showed that pancreatic or liver cancer cells treated with the considered anti-cancer drugs and quercetin exhibit increased expression levels of p53 and cleaved caspase 3. The tumour suppressor p53 is a cell cycle checkpoint protein that contributes to the preservation of genetic stability by mediating apoptosis in response to DNA damage [36]. Furthermore, the presence of caspase 3, in the cleaved isoform, clearly demonstrates an increased pro-apoptotic effect of the combination of quercetin with gemcitabine or doxorubicin on either pancreatic or liver cancer cells respectively, in comparison to the treatment with the considered anti-cancer drug alone.

It should be noted that the efficiency of the different studies drugs, used either alone or in combination with quercetin and for a given concentration, is always lower in 3D than in 2D culture. This is linked to a decreased accessibility of the considered drug to its target cell in the 3D environment. In this point of view, computational studies have explained the specific features of 3D cultures and identified several factors, which are involved, like drug diffusivity [37]. In a 3D dimension, drug accessibility takes in account of broadened random walk, as initially formulated by the mathematician Polya [38]. This stochastic process decreases, in a dimension higher than 2D, the probability of the drug particle to reach, in a sustained manner, its target. On the other hand, computational studies have also highlighted the incidences of particular drug action mechanisms, which are specifically triggered by the 3D cell conditions. For instance, we observed an induction of HIF-1α expression and the combination of either gemcitabine or doxorubicin with quercetin significantly down-regulated this expression. Such process is known to contribute to the induction of a pro-apoptotic response. It has been shown that a reduction of free radicals after specific pharmacological treatment result in the inhibition of the inflammatory response [39]. In addition, a decreased level of ROS in colorectal cancer cells is known to suppress the accumulation of HIF-1*α* caused by free radicals, thereby inhibiting their development [11]. Our results suggest therefore that quercetin could suppress ROS-induced production in 3D cultures of AsPC-1 and HepG2 cells. This could explain the suppression of HIF-1*α* expression in these studied cell models.

In conclusion, our present data demonstrate that the combination treatment of quercetin with a specific anti-cancer drug induces an increased pro-apoptotic process in both pancreatic and hepatic cancer cells, indicating that this therapeutic strategy can develop a potential response. It is therefore recommended to study, in a meet step, natural and non-toxic MDR1 and HIF-1α blockers, which are able to potentialize the efficacy of anticancer drugs in cancer cells, especially of pancreatic or hepatic origin.

## Supporting information

S1 Fig (TIF)

S2 Fig (TIF)

S3 Fig (TIF)

S4 Fig (TIF)
