# Supplementary figures and images for: Quercetin potentializes the respective cytotoxic activity of gemcitabine or doxorubicin on 3D culture of AsPC-1 or HepG2 cells, through the inhibition of HIF-1α and MDR1
Source: PLoS One. 2020 Oct 14;15(10):e0240676. doi: 10.1371/journal.pone.0240676 (PMC7556446; doi:10.1371/journal.pone.0240676)

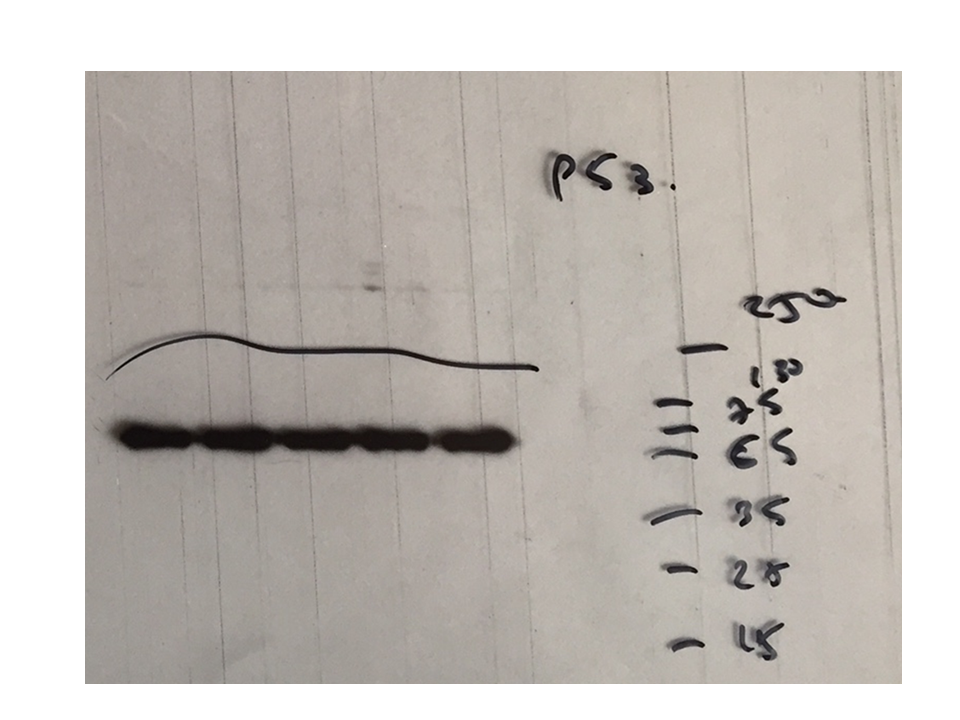

Supplement: S1 Fig — (TIF) [file pone.0240676.s001.TIF]

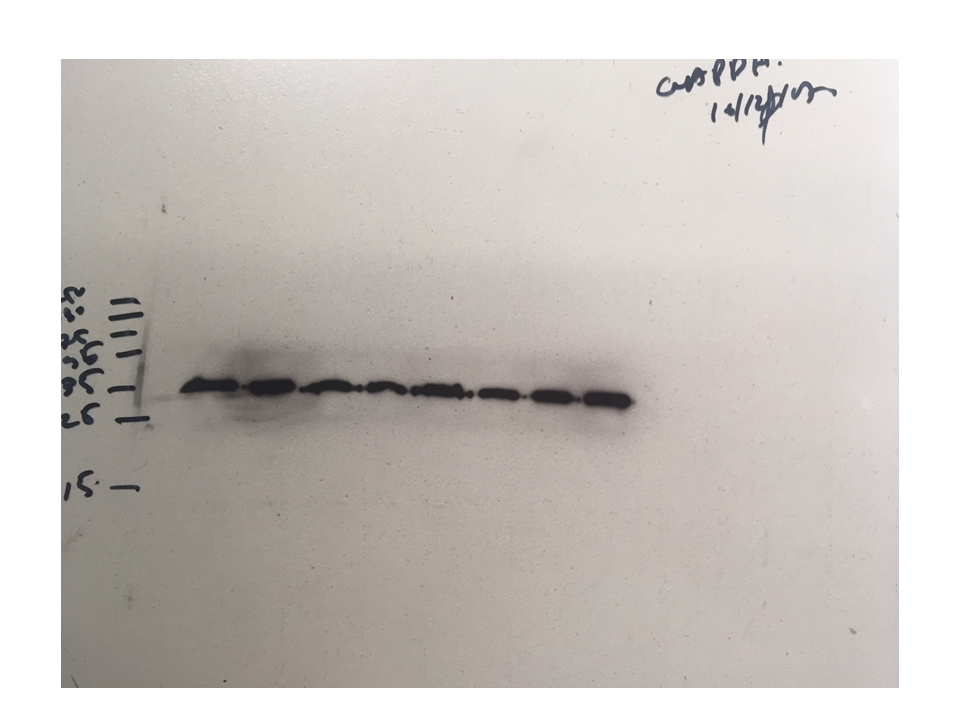

Supplement: S2 Fig — (TIF) [file pone.0240676.s002.TIF]

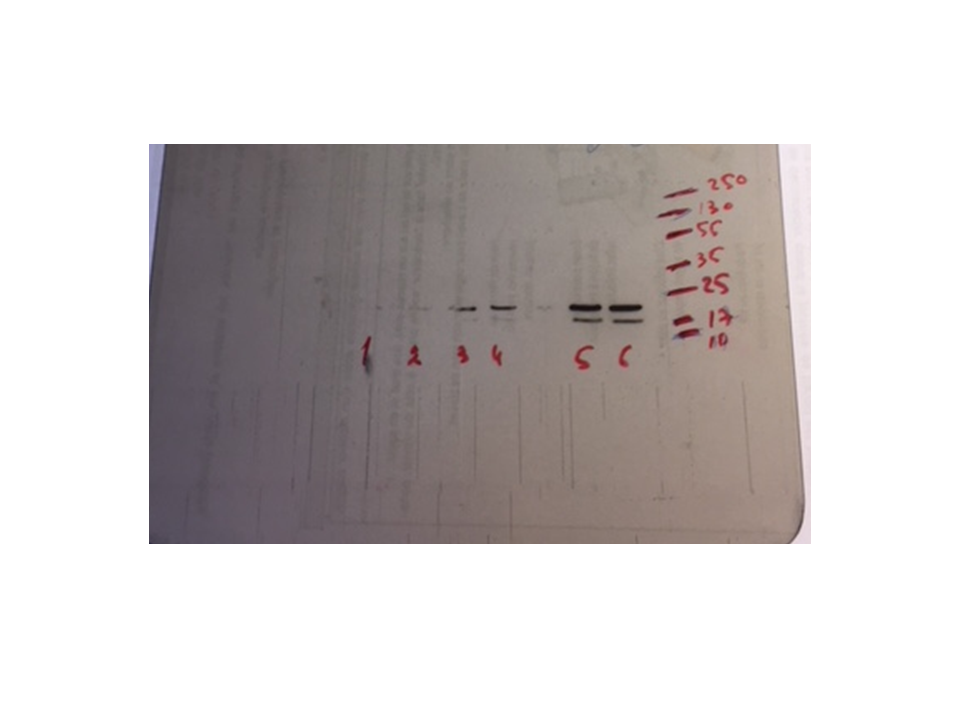

Supplement: S3 Fig — (TIF) [file pone.0240676.s003.TIF]

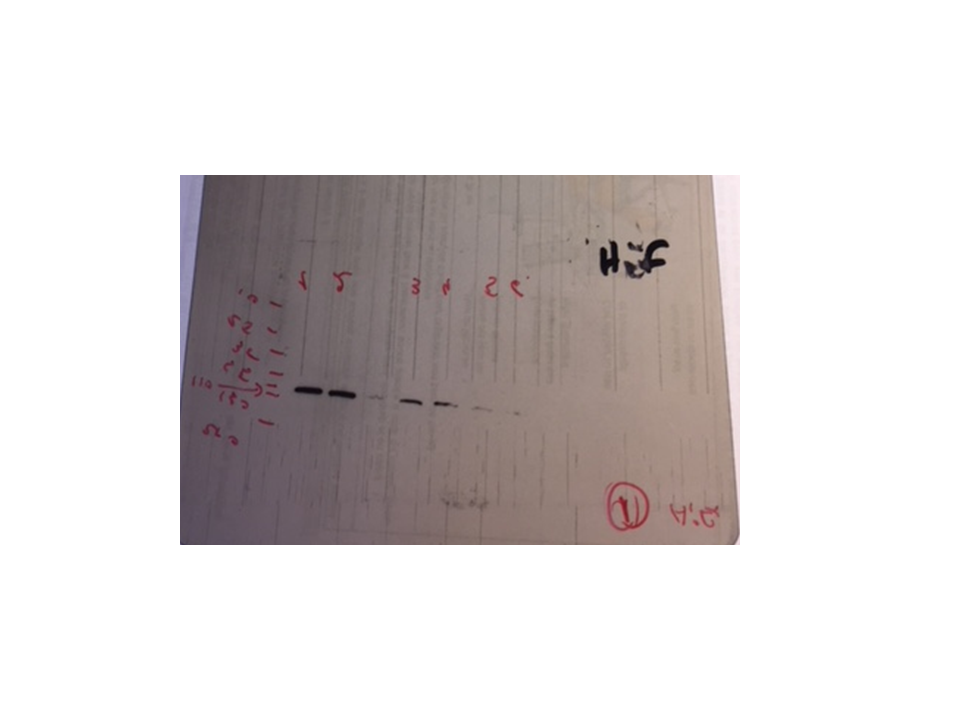

Supplement: S4 Fig — (TIF) [file pone.0240676.s004.TIF]
